# Premixed LPG + Air Combustion in a Bubbling FBC with Variable Content of Solid Particles in the Bubbles

**DOI:** 10.1007/s10494-018-9925-3

**Published:** 2018-05-07

**Authors:** Jerzy Baron, Witold Żukowski, Przemysław Migas

**Affiliations:** Faculty of Chemical Engineering and Technology, Cracow University of Technology, ul. Warszawska 24, 31-155 Kraków, Poland

**Keywords:** Fluidized bed, LPG, Combustion, Solid particles content

## Abstract

This paper presents the results of studies on the combustion of gaseous LPG in a bubbling fluidized bed. Relationships between the temperature, the bed mass and the location of the combustion zone and the NO_x_ and CO concentrations in exhaust gases are described. The concentrations of both gases increase with rising temperature and then quickly decline. It has been shown that despite the increase in average bed temperature the drop in the emission of nitrogen oxides is connected with lower temperatures inside the exploding bubbles. These temperatures strongly depend on the quantity of solid contained in them. The paper also presents the results of modeling the combustion process in a fuel-air bubble. The modeling carried out has shown that above the temperature at which bubble self-ignition becomes possible inside the bed, with further bed temperature rise there is an increase in the solids content inside the bubbles at the moment of explosion. As a result, the maximum temperature inside the bubbles falls and the emission of nitrogen oxides is reduced. In turn, the emission of CO is linked to the propagation of combustion between bubbles when self-ignition cannot take place inside them.

**Graphical Abstract:**
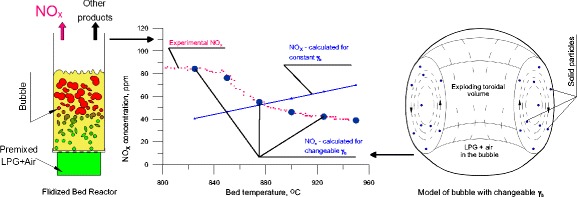
Comparison of experimental and calculated NO_x_ concentration, as a function of the fluidised bed temperature

Comparison of experimental and calculated NO_x_ concentration, as a function of the fluidised bed temperature

**Highlights**
1.: A gaseous fuel burns in a bubbling fluidized bed
2.: The combustion is intermittent and takes place inside bubbles, the combustion process starts in the toroidal part of the bubble
3.: The NO concentration is linked to the bubble temperature, not to the bed temperature
4.: The solids inside a bubble affect its thermal capacity
5.: Consequently NO concentration falls with rising bed temperature

## Introduction

In a fluidized bed, fuels can be burned in all three physical states [1]. In the case of liquid [2, 3] or solid fuels [4–8], the processes is always accompanied by the combustion of the gaseous products of their evaporation or/and decomposition [2–8]. A fluidized bed reactor can be fed, also in a specific bubbling fluidized bed combustor employing the jet-fountain configuration [9, 10].

Fluidized bed combustion of gaseous fuels is accompanied by phenomena which help in describing this process more precisely. These include dynamic effects such as pressure pulsations, optical effects (brief increases in the glow intensity - flashes) and temperature pulsation. Such phenomena appear since the combustion of a gaseous fuel mainly proceeds in bubbles and only to a small extent in the emulsion phase [11, 12]. Studies of combustion of a gaseous fuel in a fluidized bed involve reactions inside bubbles of fuel-air mixture with air, as well as burning above the bed.

After the ignition of a fuel-air mixture above a fluidized bed at ambient temperature, the mixture burns with a flame over the solids layer, constituting, in the initial stage of the process (while the bed heats up), a sort of secondary distributor with a dynamically variable surface [13]. From the bubbles reaching the bed surface some of the bed particles are thrown over into the so-called dilute phase. Since in this zone the combustion process is in progress, they are heated in the flame and then fall down on the bed surface, causing a systematic increase in the bed temperature. This facilitates the ignition of the combustible mixture, and consequently the movement of the gas combustion zone into the interior of the fluidized bed. Davidson and Hesketh [12] have reported that in the case of methane and propane combustion, the process proceeds inside the bed when the bed temperature exceeds 915 °C and 835 °C, respectively. The temperature of this transfer can be observed and is usually defined as the critical bed temperature [14]. According to the above authors, below the critical temperature the gas burns in the over-bed space.

Above the critical temperature, the transfer of the combustion process inside the bed does not imply that its course is uniform within the whole volume of the layer of solid particles. The combustion of gas in a bubbling fluidized bed is really intermittent and occurs in small, isolated elements (bubbles). This is confirmed, among other effects, by the character of the noise accompanying the process [15–19]. Combustion of the mixture occurs inside bubbles at a height from the gas distributor less than the dynamic height of the fluidized bed. Examination of the temperature distribution along the vertical axis of the fluidized bed, obtained by simultaneous measurements from of an array of a set of thermocouples, has given a stable temperature profile, while the temperature difference between layers at the highest and the lowest temperatures can reach up to about 50 °C [20]. The location, in relation to the distributor, of the layer at the highest temperature, H_Tmax_, depends on the average bed temperature T_bed_ (in a bed with a higher average temperature, the hottest region is closer to the distributor), the type of gaseous fuel burned and other factors.

The discontinuous character of the combustion, located in a relatively narrow layer, also appears to be confirmed by observations and measurements on temperature oscillations within the bed. Largest oscillations (amplitudes of 5-10 K) occur in the zone at the highest temperature [19]. Oscillation with such a large amplitude can result from a violent “pushing” of the gaseous products into the emulsion, after explosions of the fuel-air mixture inside bubbles causing a brief rise in the temperature of the near-by thermocouples.

The use of a photomultiplier to monitor light emission with simultaneous sound recording has shown that the flashes are always accompanied by strong sound impulses [21, 22]. Żukowski has found that when the bed temperature rises three successive states can be distinguished in the photometric signal from the bed surface [21].

The first one (below T_CR1_) has the characteristics of noise, the second state can be seen, in which, in addition to the noise, distinct light high amplitude signals appear and above T_CR2_ the third state appears, with noise signals dominant - no peaks due to light impulses occur in this state since the bubbles explode inside the bed.

Temperatures, at which the type of photometric records changes are defined as two critical bed temperatures [21]. The second one should be identified with the critical temperature as defined by Davidson [12] or Baskakov [14].

In the case of a bed with a mass of 300 g (i.e. dynamic height 80 mm at 900 °C) - enabled us to obtain critical temperatures for methane T_CR1_ = 550 °C; T_CR2_ = 860 °C, ethane T_CR1_ = 525 °C; T_CR2_ = 808 °C [21] and LPG T_CR1_ = 535 °C, T_CR2_ = 830 °C. Similar information could also be obtained in the case the addition to LPG of such components as dichloromethane (DCM) or chlorobenzene [23].

Using model calculations, it has been shown that only above the second critical temperature ignition in bubbles can be kinetically controlled [19]. The position of exploding bubbles and consequently the location of the combustion zone result from the velocity of their movement through the bed and the time needed for ignition inside them. The calculations carried out have shown that the gases fed through the distributor to the bed quickly achieve a temperature equal to that of the emulsion and at that temperature they move up the bed. At the moment of ignition the next, abrupt increase in the gas temperature in the bubbles occurs, followed by its gradual drop due to heat exchange between the post-reaction gases and the emulsion. The sharp increase in temperature also results in a pressure rise that causes the acoustic waves [18, 21].

An interesting relationship observed during the combustion of fuels in various physical states in a fluidized bed is associated with the formation of nitrogen oxides. When gaseous fuels are burned, the concentration of NO_x_ in the post-reaction gases is connected with the position of the combustion process in relation to the surface of the fluidized bed and the combustion mechanism inside the bubbles. Nitrogen oxides appear in the gases above the bed in all the three ranges of bed temperature, separated by the two critical temperatures. Their formation, with no fuel nitrogen, is explained by the mechanisms proposed by Zeldovich [24] and by Fenimore [25]. It is known that with both these mechanisms, as the temperature in the combustion zone rises, the concentration of NO_x_ in exhaust gases always increases.

The most important observation is, that in the case of combustion in fluidized beds, relationships apparently incompatible with the above general experience can be seen. In the first and second range of bed temperatures (i.e. below the second critical temperature) the NO_x_ concentration in the off gases increases with rising temperature, but above the second critical temperature, in many cases, a drop in the NO_x_ concentration was observed [18, 21, 23]. In what follows we shall try and explain this since this relationship runs against the usual argument based on chemical kinetics. We shall also examine the dependence of the CO concentration on temperature characterized by a maximum within the range (T_CR1_ T_CR2_) [19, 23], and also try to find possible reasons for it.

## Experimental Setup

Tests were carried out using a cylindrical quartz fluidized bed reactor with an internal diameter of 98 mm and height of 500 mm. The distributor was made of chromium-nickel steel plate with some 433 holes (diam. 0.6 mm, 4 mm apart) in a square array (free area 1.8%). In our experiments the bed used consisted of 100, 200, 300 or 400 g of quartz sand of particle size 300–385 μm. These masses correspond to the static heights of the bed amounting to 9, 18, 27 and 36 mm, respectively.

**Fig. 1:**
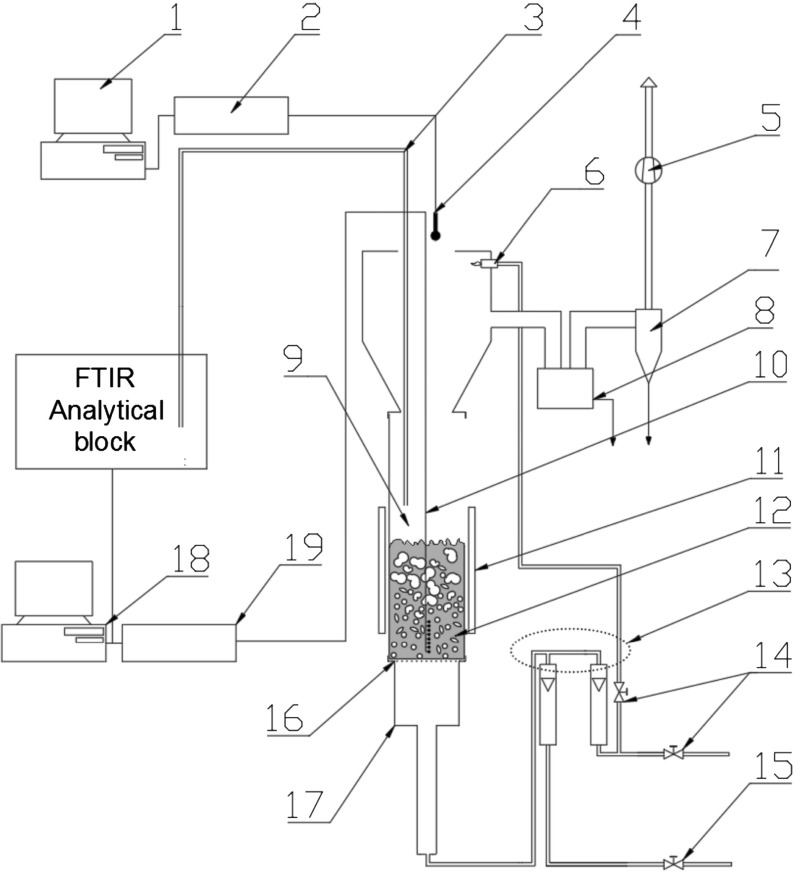
Experimental arrangement - schematic representation. 1 – computer storing the acoustic data, 2 – A/D converter for acoustic signals, 3 – heated probe for sampling the flue gases, 4 – microphone, 5 – exhaust fan, 6 – pilot flame, 7 – cyclone, 8 – ash trap for coarser particles, 9 – freeboard space, 10 – set of 8 thin thermocouples - vertically mounted, 11 – movable radiation shield, 12 – bubbling bed, 13 – mixing node, 14 – LPG supply valves, 15–Air supply valve, 16 – flat, perforated metal plate distributor, 17 – plenum chamber, 18 – computer storing chemical analysis results and temperature signals, 19 – A/D converter for thermocouple signals

In order to determine the vertical temperature profile within the bed, a system of eight Ni-NiCr thermocouples was constructed. The thermocouples were located at heights of 7, 12, 20, 30, 40, 50, 60 or 70 mm above the distributor. The sampling frequency of temperature signals was 10 Hz Uncertainty in temperature measurement was 1 K. The flue gases were analysed using a FTIR Gasmet^TM^ DX-4000 analyser. The uncertainty in NOx and CO concentration measurements were 0.5 ppm. A schematic diagram of the test stand is presented in Fig. 1.

Air, with at a flow rate of V_air_ = 1.66 L s^− 1^ and at a temperature of about 20 °C, mixed with gaseous LPG at a flow rate of V_LPG_ = 0.033 L s^− 1^ were supplied to the plenum chamber and then through the distributor to the bed. The combustion of LPG mixed with air took place with the excess air coefficient *λ* of 2, which means that the air flow is twice as large as the stoichiometric flow resulting from the amount of the supplied fuel. The mass of the sand bed and the bed temperature constituted independent variables, while the dependent variables included the concentrations of selected components in the flue gases, fluctuations in temperature and acoustic pressure in the freeboard space and the dynamic height of the bed.

After the fuel-air mixture was ignited, the rate of heat exchange with environment was controlled by means of a movable shield around the reactor. During a typical run the average bed temperature was increased from ambient to about 1000 °C and then the reactor wall was slowly cooled until the bed temperature fell to slightly above 700 °C Then it was reheated again to about 1000 °C. After such a cycle, the gas supply to the reactor was turned off, the reactor was cooled and 100 g of sand was added. This caused a rapid temperature drop (to below 700 °C). Then the flow of the fuel-air mixture was turned on and the gases re-ignited.

## Results and Discussion

### The combustion process

**Fig. 2:**
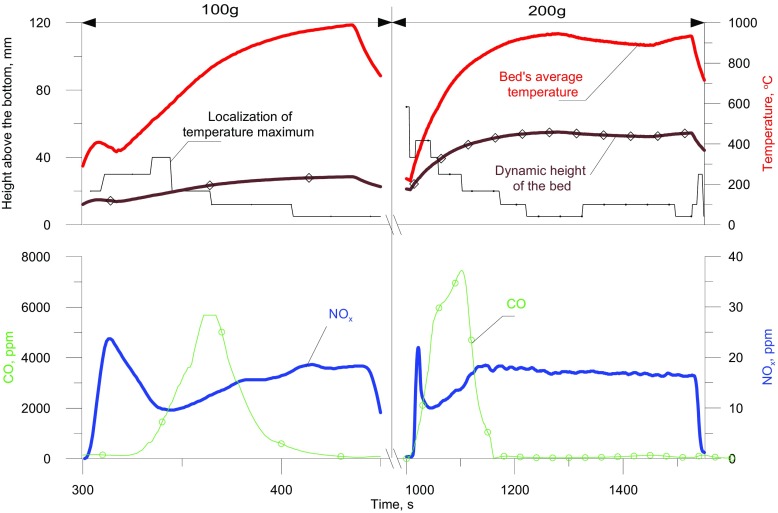
Changes in the temperature, calculated bed height, the position of the zone at the highest temperature, and concentrations of NO and CO. Bed mass = 100 or 200 g

**Fig. 3:**
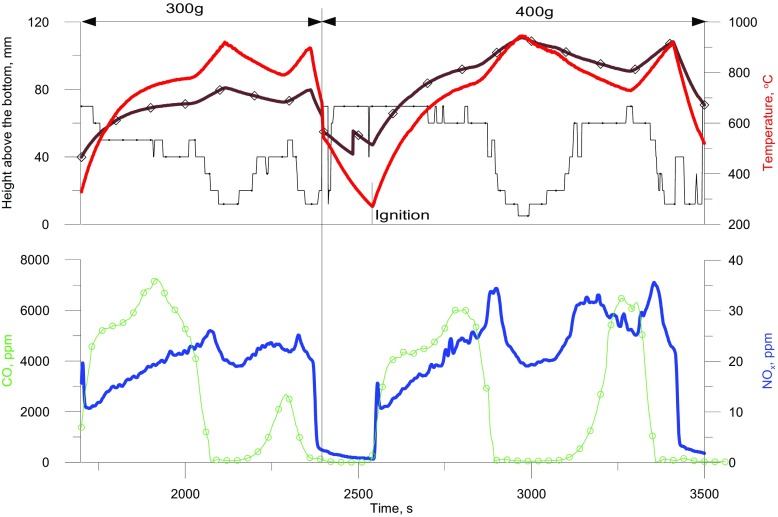
Changes in the temperature, calculated bed height, the position of the zone at the highest temperature, and concentrations of NO and CO. Bed mass = 300 or 400 g (Description of the plots as in Fig. 2)

An increase in the mean bed temperature leads to a rise in the temperature of the fuel-air mixture in the bubbles and in the emulsion. This leads to increasing gas flow rate through the bed and consequently to increasing voidage and bed height. Changes in the mean temperature of the bed and its height and the position (in relation to the distributor) of the zone in the bed where the maximum temperature occurs are presented in Figs. 2 and 3. Within the range of 20950 °C, there is a considerable increase in the bed height. For example, at 90 °C the dynamic height of beds with 100, 200, 300 and 400 g of bed material amounted to 26, 54, 80 and 105 mm, respectively. Changes in the NO_x_ and CO concentrations in flue gases are also shown in Figs. 2 and 3.

It is a characteristic of beds of various heights that, the crossing of the lines showing the dynamic height of the bed and the position of the zone with the highest temperature occurs at a temperature of about 600 °C. This is in effect when combustion moves from above to just under the surface of the bed (transition from the first to the second phase of combustion) when reaction propagation between large bubbles dominates and the temperature of the solids is decisive important With increasing bed mass (and dynamic height) this temperature is insignificantly reduced. These facts underline the essential importance of T_CR1_ in the description of gas combustion in a fluidized bed. During the combustion of gaseous LPG the concentrations of CO and NO_x_ in the flue gases were dependent on both the position of the combustion zone and the average bed temperature.

Based on the indications of thermocouples coaxially located in the bed, the mean bed temperature T_bed_ was obtained and the position of combustion zone determined. The mean bed temperature was taken as the average temperatures indicated by 5 thermocouples immersed in the bed, omitting the one nearest to the distributor and two uppermost ones located at 60 and 70 mm above the distributor. This limitation results from the fact that the cooler distributor affects the temperature measurement from the thermocouple next to it. In the case of the two uppermost thermocouples, during measurements they can occasionally be above the fluidized bed surface.

**Fig. 4:**
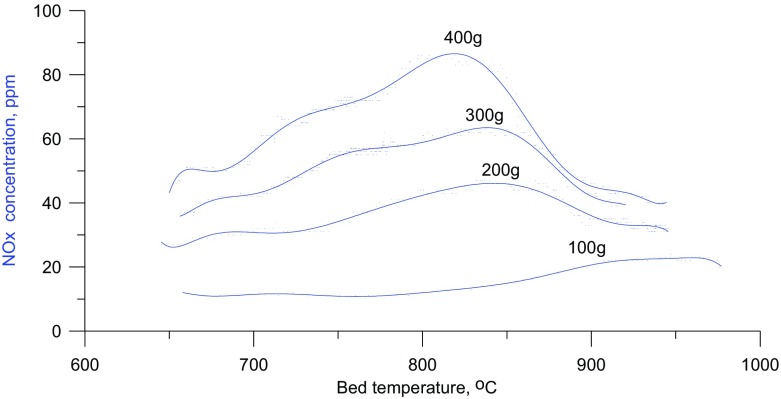
Concentration of nitrogen oxides in the exhaust gas depending on the mass and the average temperature of the bed

With rising bed temperature the NO concentration changes characteristically (Fig. 4). At a given temperature smaller quantities of NO are formed in a shallower bed than in a deeper one. With increasing bed temperature, until the second critical temperature is reached, the NO_x_ concentration in the flue gases rises, which results directly from the dominance, in this temperature range, of the “prompt mechanism” of NO formation. This happens in shallow beds (e.g. 100 g bed, H_d_ = 26 mm) as well as in higher beds (e.g. 400 g bed, H_d_ = 105 mm). Unlike in the case of gaseous fuel combustion on a burner, in a fluidized bed above the second critical temperature, the tendency of the NO concentration to grow with increasing temperature halts. This is followed by a drop in the NO concentration in the post-reaction gases, the earlier, the higher the bed. The previous combustion model, in the range in which T_bed_ > T_CR2_, does not predict this dependence since, according to that model, the concentration of NO should still increase with rising temperature.

The limited rate of heat exchange between the gas inside bubbles and the emulsion phase in the bed is responsible for the thermal energy produced when the mixture explodes in a bubble brings about a rapid rise in the temperature of the gases inside the bubble. This also causes an increase in the quantity of NO formed. Just after the ignition of the fuel mixture in a bubble the gas temperature only weekly depends on the temperature of the surrounding emulsion, while an increase in the emulsion temperature leads to an increase in the maximum temperature inside a bubble after ignition. Measurements show that this does not translate into further increase in the NO concentration in the product gases.

### Results from the model

Mathematical models of fluidized bed combustion known from the literature are concerned both with the interior of the bed and the freeboard zone [26, 27]. The modification of the model developed by Żukowski [19] was proposed for calculations on the combustion of the fuel mixture inside bubbles. It should be emphasized that, as in the model described earlier [19], the mass and energy balance equations do not refer to the entire bubble but to its selected toroidal part. This is due to the fact that, depending on the velocity at which bubbles move in the bed, the gas movement within the bed is shaped accordingly. According to the proposal of Kunii and Levenspiel [28] the ratio of bubble flow velocity u_br_ to the u_mf_/ε_mf_ ratio should be taken into consideration. When the ratio is greater than 1, as in the case discussed here (precise calculation of u_br_/(u_mf_/ε_mf_) is included in the Appendix), gas in the bubble circulates and its part circulates without contact with the emulsion zone.

The present model, describing gas combustion in a fluidized layer, is based on the kinetic model of Konnov [29] and the model of bubble growth of Mori and Wen [30]. This model has been recently used by Menon et al. [31], but they also assumed that the combustion of the vapours of a hydrocarbon fuel occurs in the bubbles, and in fact in the thoroidal part, where the gas circulates without contact with the emulsion. In the model presented here the fuel-air bubbles in which combustion occurs, are treated as non-isothermal reactors containing a mixture of the gaseous fuel with an oxidizer in the presence of solid particles. Changing the organization of gas combustion in fluidized bed, Zeidan and Okasha [32] has shown how significant for heat exchange in a fluidized bed is the transfer of a part of the bed material to the above-bed zone. Among the effects of changing the organization of the combustion process observed by them was the homogenization of the fluidization process through the decreasing size of bubbles and reduction in NO_x_ emission and in the level of noise. The modification of the model mentioned above consists in taking into account the effect of bed particles on the course of the burning of gaseous fuels in the bubbles, assuming that the volume fraction of solid particles in the bubbles changes. This assumption is physically justified, taking into account the transformations of bubbles. These are formed in the emulsion at the perforated distributor at the bottom, but there is no analogy to the formation of bubbles in a liquid, since there is no phase boundary and consequently no surface tension at it (interactions of sand particles with a diameter of a hundred micrometers in a fluidized bed can be omitted, as this material falls in area B according to Geldard’s classification [33]). Hence, the bubbles being formed will contain some solid material. As bubbles move through the bed, the emulsion can not act directly on the solid contained in them, so gravitation must bring about gradual loss of particles from the bubbles. Additionally, an increase in the bubble volume can give an identical effect. Therefore the content of solids in the bubbles should be a decreasing function of their distance from the distributor.

In model calculations, 128 differential equations were solved, using the Konnov kinetic model [29] and taking into account the mass balance of 127 chemical species and the heat balance. The mass balance for the i-th component was expressed by Eq. 1:

1 $$ \rho_{g} \frac{dx_{i}} {d\tau} =\sum\limits_{j = 1}^{m} {r_{i,j} M_{i} {\begin{array}{*{20}c} \hfill & \hfill & \qquad{i = 1\,} \hfill \\ \end{array}} .....\,n} $$

Expressions describing the gas mass transfer between bubbles and the emulsion have been omitted from this equation. This is due to the fact that in the discussed example u_br_ to (u_mf_/ε_mf_) ≈ 7.8 (at 800 °C), which quite well corresponds to the case described by Kunii and Levenspiel [28] in chapter 5, Fig. 3e, according to which in the bubble one part of gas may be distinguished which, by circulation, periodically comes into contact with the external space (and in such a way the mass transfer with the surroundings takes place) and the other part of gas which, circulating, remains within the bubble all the time. In the external part the circulating gas exits the bubble, passes through the cloud-and-wake zone and then returns to the bubble. In the internal part the gas basically remains in the bubble all the time and has no contact with the cloud-and-wake zone. In the modelling of catalytic processes taking place in the emulsion, the mass transfer between the emulsion and the bubble plays a crucial role and is physically realized through the external part of the circulating gas presented in Fig. 3e [28]. However, in the case of the combustion process of a premixed gaseous mixture (fuel-air) in the bubble the external part of the mixture may be omitted in an analysis of explosive phenomena since periodic gas immersion in a solid-rich environment leads to the recombination of radicals and phasing out the developing radical processes. In the bubble toroidal zone assumed as a control volume, which may be treated as a perfectly mixed reactor with no contact with the surroundings, the development of radical processes takes place, which in turn leads to a rapid increase in temperature and pressure, i.e. to an explosion of a combustible mixture in the bubble. As a result, gases from the toroidal zone mix with the bubble external zone leading to the combustion of the combustible mixture residue, and then form an acoustic wave and transfer heat to the emulsion.

In the heat balance equation not only the thermal effects of particular chemical reactions were taken into account but also the heat exchange between a bubble and the emulsion phase and that between gas and the solids present in a bubble:

2 $$ \rho_{g} C_{pg} \frac{dT_{b}} {d\tau} +\sum\limits_{i = 1}^{n} {\left( \sum\limits_{j = 1}^{m} {r_{i,j} M_{i} h_{i}} \right)+(\alpha_{g}^{bc} +\rho_{s} c_{s} \beta_{s}^{bc} )} (T_{e} -T_{b} )= 0 $$

Where $\alpha _{g}^{bc} $ is the coefficient of thermal conductivity between bubble and emulsion and it is defined by the expression [28]:

3 $$ \alpha_{g}^{bc} = 5,85\frac{g^{0,25}\sqrt {k_{g} \rho_{g} C_{pg}} } {d_{b} (h)^{1,25}} $$

In Eq. 2 the coefficient $\beta _{s}^{bc} $ is connected with the motion of solid particles inside a bubble and is defined by the equation proposed by Essekat and Tabiś [34]:

4 $$ \beta_{s}^{bc} =\frac{3u_{t} \gamma_{b}} {d_{b} (h)} $$

The modeling involved determining the effect of the content of solids in a bubble and the temperature of the emulsion phase in the bed on the temperature of the gases in an exploding bubble, the concentration of nitrogen oxides formed and the induction time for auto ignition.

**Fig. 5:**
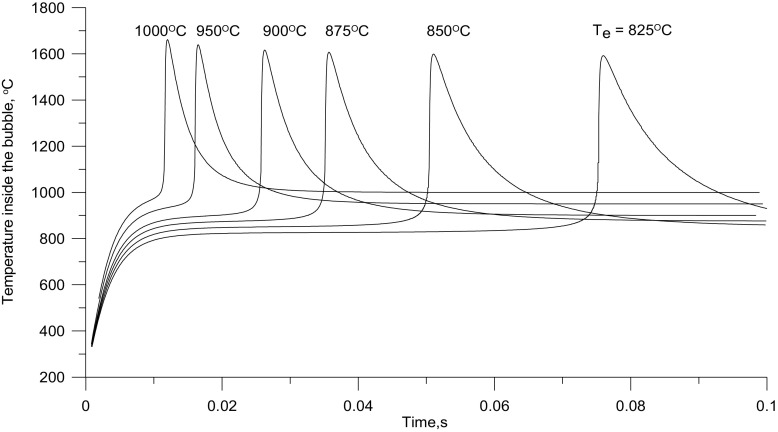
Calculated changes in the bubble interior temperature for different bed temperatures and a constant content of solid particle of 0.3%

The results of the first series of calculations carried out for a constant content of solids in a bubble of 0.3%_vol._ are presented in Fig. 5. The initial temperature of bubbles assumed for all calculations amounted to 20 °C.

The bubbles moving up in the bed are heated towards the temperature of the bed particles. As the bubbles travel through the bed, the rate of temperature rise in their interior decreases due to falling temperature difference between the bubble interior and the bed. At the end of the induction time, ignition of the gases inside a bubble leads to a rapid rise in their temperature. Once the combustion is over, the temperature of the gaseous products begins to fall towards the emulsion temperature. An increase in the average bed temperature leads to shorter induction times for ignition and slightly higher maximum temperature inside the bubble due to the explosion of the gas in it. The maximum temperature obtained from the model with the emulsion at 1000 °C is just under 1700 °C. Similarly, in ref. [27] modelling was used to show that the interior of a bubble temperature in which combustion occurs is appreciably higher than that of the emulsion. The temperature rise inside bubbles is important for the formation of NOx.

**Fig. 6:**
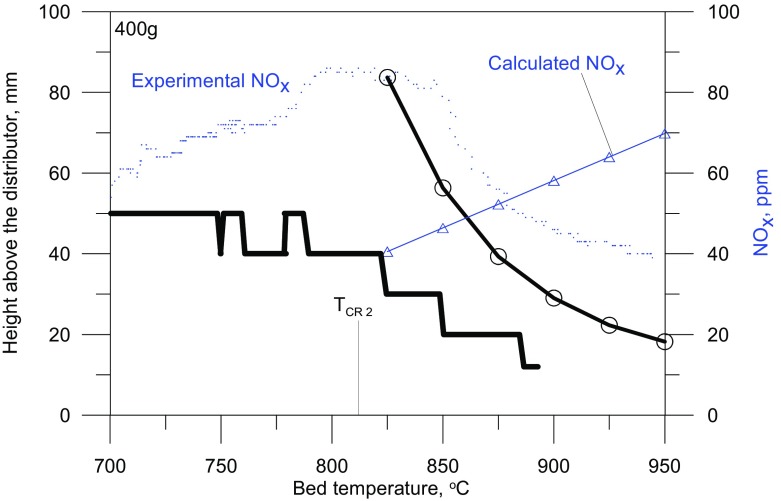
Location of the temperature maximum in the vertical profile of the reactor, calculated and experimental NOx concentrations, and the calculated position of bursting bubble center - dependence on the average bed temperature

As is shown in Fig. 6, the assumption that a bubble contains a constant volume fraction of solid is not strictly true, although the induction time corresponds to the experimental data, under such conditions the concentration of NO_x_ increases with the bed temperature.

**Fig. 7:**
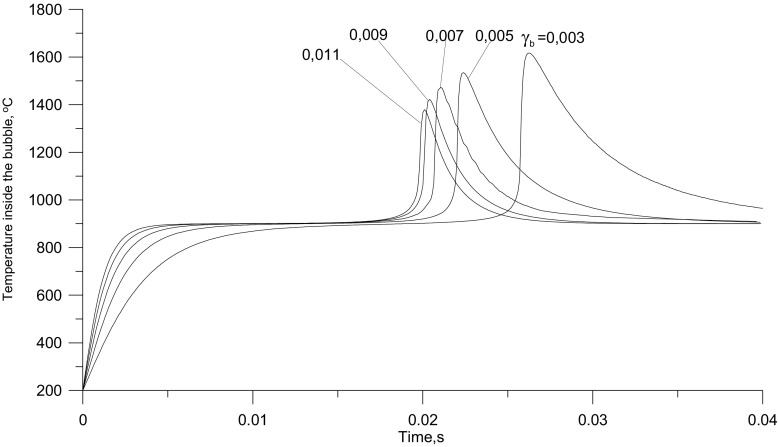
Calculated changes of the temperature in the bubble interior, for the different solid particle content and the bed temperature constant at 900 °C

A change in the content of solids in a bubble very significantly affects the course of the combustion process in its interior. The solid particles inside a bubble play a double role. On the one hand they are a source of heat for the gases present inside the bubble, especially in the initial phase of its travel up the bed. On the other hand, when their temperature is lower than the temperature inside the bubble, they can have a cooling effect. For example, for mean bed temperature of 900 °C, increasing the solid content in a bubble shortens the induction time for ignition, but – after the ignition of the mixture – it leads to a decrease in the maximum temperature in its interior. Figure 7 shows the dependence of temperature changes inside a bubble as a function of time, calculated, assuming a constant temperature of the emulsion phase in the bed (900 °C) and the content of solid particles in the bubble variable within the range of 0.3–1.1%_vol._

**Fig. 8:**
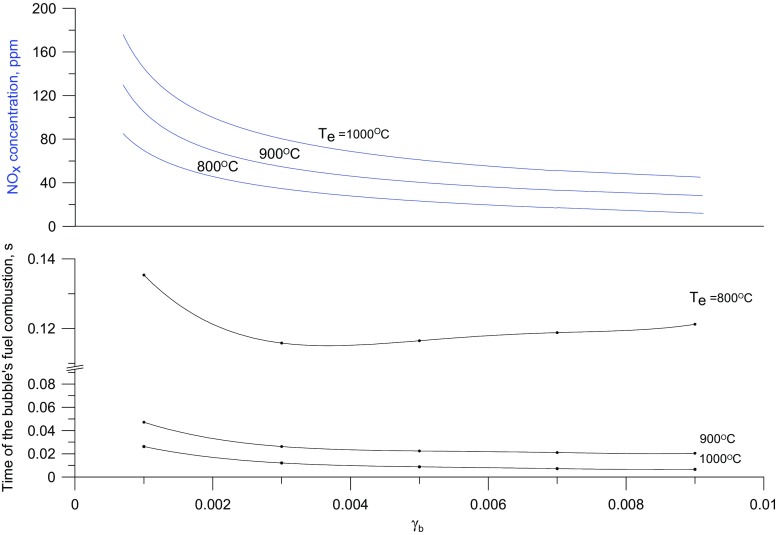
Calculated NOx concentration and combustion time for the fuel inside bubbles as a function of the solid particle content in them (*γ*_*b*_), for different bed temperatures

It seems obvious that an increase in the solid content increases the quantity of heat energy supplied to bubbles, and consequently increases the initial rate of temperature rise inside bubbles and shortens the time needed to reach the maximum temperature (i.e. time needed for the reaction to run to completion). When a certain level of solid content is exceeded, a reverse process may dominate, i.e. bubble chilling with the slowing down of the combustion reaction. This effect should become more important with lower temperatures of the bed emulsion. This implies that there should be a solid content, such that at the given bed temperature, there will be a local minimum time for the completion of the combustion of fuel in bubbles. For bed temperature 800 °C and solid content of about 0.3%_vol.bj._ this minimum was calculated (Fig. 8). The modeling results show that the temperature of the post-reaction gases inside bubbles can reach 1700 °C.

**Fig. 9:**
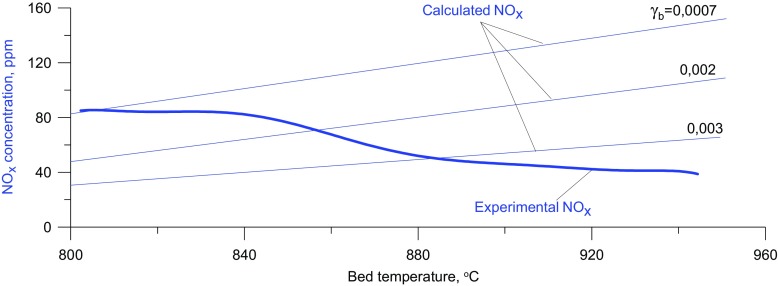
Experimental (400g bed), and model (variable solid particle content) NOx concentration

Figures 8 and 9 illustrate the effect of the bed temperature and the content of solids on the concentration of nitrogen oxides in the off-gases. It has been found that an increase in the temperature of the gas leads to a rise in the calculated NO content in the gases. Thus the responses of the model are consistent with observations made using gas burners. It can also be seen that with increasing solid content in the bubbles the concentration of NO_x_ decreases. This results from the slowing down of the oxidation of atmospheric nitrogen because the temperature of the gases inside bubbles is lowered by the bed particles. Another reason for it is that the concentration of free radicals involved in the formation of NO falls when the effective surface of the bed particles on which their recombination can take place increases.

A decrease in the emission of NO_x_ observed after the second critical temperature is exceeded implies that with increasing bed temperature, and hence with smaller dimensions of exploding bubbles, the temperature inside a bubble at the moment of explosion is actually falling The maximum temperature reached by the gas in a bubble exploding closer to the distributor is lower than that of one exploding in the vicinity of the upper bed surface. On the basis of the results for the modified model, it follows that the drop in the maximum temperature inside a bubble is due to an increase in the content of solid present in it. The solids content in an exploding bubble, at a given temperature, can be determined as the point of intersection of the experimental curve representing the NO_x_ concentration as a function of temperature, with the curve obtained by modeling with a specified content of solid in the bubble (Fig. 9).

**Fig. 10:**
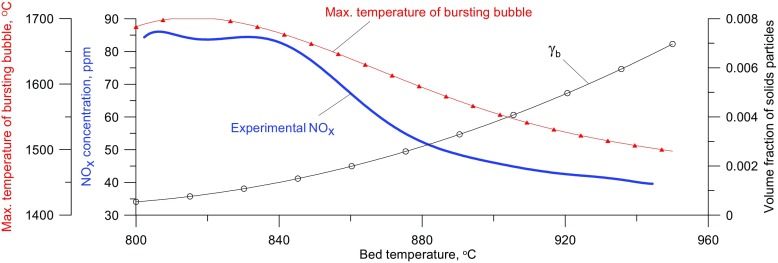
Experimental NOx concentration, solid particle content, and the max bubble interior temperature during its explosion, as a function of the mean bed temperature

**Fig. 11:**
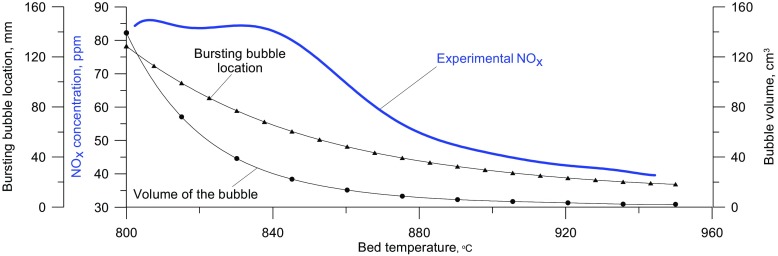
Experimental NOx concentration, volume and location of a bursting bubble as a function of the mean bed temperature

The results of the calculations carried out indicate that within the bed temperature range from 820 °C (T_CR2_ for the bed with a mass of 400 g) to 940 °C the distance of the bubble center from the distributor at which the gas contained in the bubbles explodes changes from about 95 mm to about 20 mm. At the same time, the volume of these bubbles decreases from about 60 cm^3^ to almost 1 cm^3^, while the volume fraction of the solid contained in their interior at the moment of explosion increases from 1‰ to 7‰. This causes a drop in the maximum theoretical temperature reached inside the bubbles from about 1700 °C to 1500 °C, resulting in a NO_x_ concentration in the off gases lower by about 40 ppm (Figs. 10 and 11).

**Fig. 12:**
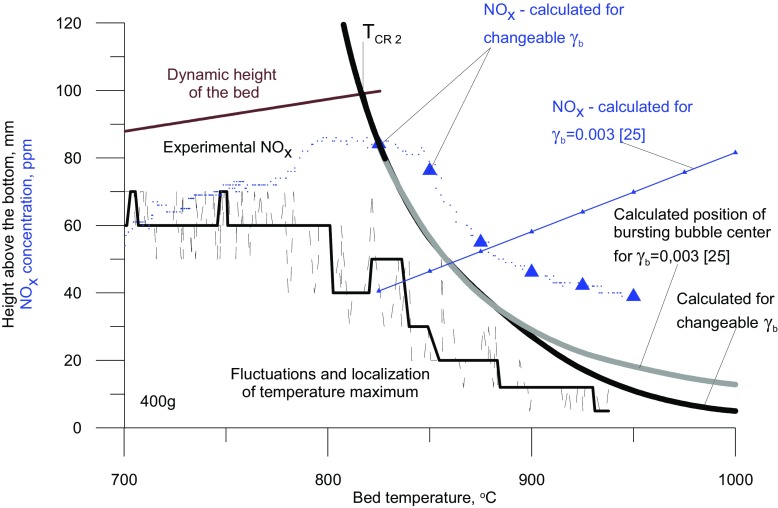
Height of the bed, location of temperature maximum in the vertical profile of the reactor, NO_*x*_ concentrations and calculated position the centre of a bursting bubble – dependence on the mean bed temperature

The addition to the model of an additional variable, i.e. the content of solids inside bubbles makes it possible to describe precisely the gas combustion process of in a bubbling fluidized bed. As seen in Fig. 12, for variable values of *γ*_b_, the calculated locations of exploding bubbles are more precisely fitted to the experimental results, especially in the area close to the distributor. Where T_bed_ > T_CR2_ the NO_x_ concentration derived from the model, taking into account the variability of the content of bed particles inside bubbles, decreases with increasing mean bed temperature, which corresponds to observations.

## Conclusions

A significant effect occurring during the combustion of a gaseous fuel in a bubbling fluidized bed is the presence of a zone of maximum temperature inside the bed. It is the ignition induction time of the fuel mixture in bubbles, dependent on the temperature of the reagents that constitutes a factor determining the location of this zone in relation to the distributor. Three characteristic types or regimes of the course taken by the combustion process for LPG vapour in a bubbling fluidized bed can be identified: combustion above the bed, combustion in the near-surface space of the bed and combustion inside the bed. The transition from one regime to the next occurs at the corresponding critical temperature of the bed (T_CR1_ or T_CR2_).

During combustion, the material at the bed surface is in a continuous motion and the bed particles are thrown into the above-bed space destabilizing the combustion process in the diffusion flame above the bed surface. This slows down the combustion process in the first and second combustion regimes, i.e. with the mean bed temperature lower than T_CR2_ and leads to an increase in the CO concentration in the post-reaction gases.

The dependence of the NO_x_ concentration on temperature is characterized by the occurrence of a NO_X_ concentration maximum at temperatures about 820 °C – 850 °C. This is close to the value of the second critical temperature. Once this temperature is exceeded, a decrease in the concentration of nitrogen oxides in flue gases with increasing bed temperature can be seen. The results of modeling the combustion process for LPG inside fuel-air bubbles, taking into account the concentration of solids inside the bubbles, show that when the temperature exceeds T_CR2_, as the distance of an exploding bubble from the distributor decreases, there is a significant increase in the solid content in the bubble interior. As a consequence there is a considerable drop in the maximum temperature of the gaseous products of combustion, which results in slowing down the rate of NO_x_ formation from atmospheric nitrogen. An evident effect of this is the decrease in NO_x_ concentration in flue gases despite the rising temperature of the bed emulsion.

## Appendix

Reactor diameter D:= 0.096⋅m P$:=\frac {\uppi \cdot \text {D}\cdot \text {D}}{4}$Gas parameters

$$\begin{array}{@{}rcl@{}} \begin{array}{ll} \upeta(T)\!:=\!17.168\cdot0.000001\cdot(\text{Pa}\cdot \sec) \cdot \frac{273 \mathrm{K}+ 114\cdot \mathrm{K}}{\mathrm{T}+ 114\cdot \mathrm{K}}\cdot \left( \frac{\mathrm{T}}{273\cdot\mathrm{K}}\right)^{1.5} &\qquad\!\!\mathrm{M}\!:=\!\left( \!\begin{array}{ll} 5\cdot\%\cdot32 + 7\cdot\%\cdot44\ldots\\ + 15\cdot\%\cdot18 + 73\cdot\%\cdot28 \end{array}\!\right)\cdot\frac{\text{kg}}{\mathrm{kmo1}}\\ \mathrm{u}(\mathrm{T})\!:=\!\frac{\mathrm{T}}{(273 + 20)\cdot\mathrm{K}}\cdot\frac{(1.66 + 0.033)+\frac{\text{dm}^{3}}{\sec}}{\mathrm{P}}&\qquad\!\!\uprho(\mathrm{T})\!:=\!\frac{\mathrm{M}\cdot1\text{atm}}{\mathrm{R}\cdot\mathrm{T}} \end{array} \end{array} $$

Solids parameters

$$\begin{array}{lll} \mathrm{d}_{\mathrm{z}}:=\frac{0.385 + 0.3}{2}\cdot\text{mm}&\qquad{\uprho}_{\mathrm{z}}:= 2760\cdot\frac{\text{kg}}{\mathrm{m}^{3}}&\qquad\upvarepsilon_{\text{mf}}:= 0.45 \end{array} $$

Calculations

$$\begin{array}{@{}rcl@{}} \begin{array}{ll} \text{Ar}(\mathrm{T}):=\mathrm{g}\cdot\mathrm{d}^{3}_{\mathrm{z}}\cdot\frac{\left( {\uprho}_{\mathrm{z}}-{\uprho}_{\mathrm{g}}(\mathrm{T})\right)\cdot{\uprho}_{\mathrm{g}}(\mathrm{T})}{{\upeta}_{\mathrm{g}}(T)^{2}}&\qquad\!\!\text{Re}_{\text{mf}}(\mathrm{T}):=\left( \sqrt{28.7^{2}+ 0.0494\cdot\text{Ar}(T)-28.7}\right)\\ \mathrm{u}_{\text{mf}}(\mathrm{T}):=\frac{\text{Re}_{\text{mf}}(\mathrm{T})\cdot{\upeta}_{\mathrm{g}}(\mathrm{T})}{\mathrm{d}_{\mathrm{z}}\cdot{\uprho}_{\mathrm{g}}(\mathrm{T})}\\ \mathrm{d}_{\mathrm{b}}\!:=\!3\text{cm}\ \ \mathrm{T}\!:=\!(800 + 273)\mathrm{K}&\qquad\!\!\mathrm{u}_{\mathrm{b}}(\mathrm{T})\!:=\!\mathrm{u}(\mathrm{T})\,-\,\mathrm{u}_{\text{mf}}(\mathrm{T})+ 0.71\cdot\sqrt{\mathrm{d}_{\mathrm{b}}\cdot\mathrm{g}}\cdot1.2\cdot \exp\left( \!-1.49\cdot\frac{\mathrm{d}_{\mathrm{b}}}{\mathrm{D}}\!\right) \end{array} \end{array} $$

Results

$$\begin{array}{llll} \mathrm{T} = 1.073{\times}10^{3}\mathrm{K}& \mathrm{u}(\mathrm{T})= 0.857{\cdot}\frac{\mathrm{m}}{\mathrm{s}}& \qquad\mathrm{u}_{\text{mf}}(T)= 0.062{\cdot}\frac{\mathrm{m}}{\mathrm{s}}&\!\qquad\mathrm{u}_{\mathrm{b}} (\mathrm{T}) = 1.084{\cdot}\frac{\mathrm{m}}{\mathrm{s}}\\ \frac{\mathrm{u}_{\mathrm{b}}(\mathrm{T})}{~}= 7.809\\ \frac{\mathrm{u}_{\text{mf}}(\mathrm{T})}{{\upvarepsilon}_{\text{mf}}} \end{array} $$

## Symbols

C_pg_: – molar heat capacity of the gases at constant pressure, J mol^− 1^ deg^− 1^
c_s_: – specific heat capacity of the solids, J kg^− 1^ deg^− 1^
d_b_: – bubble diameter, m
g: – acceleration due to gravity, m s^− 2^
h: – height above the distributor, m
h_i_: – specific enthalpy of the i-th species, J kg^− 1^
H_d_: – height the bed, m
H_Tmax_: – distance of the max temperature zone from the perforated distributor plate, m
k_g_: – thermal conductivity of the gases, W deg^− 1^ m^− 1^
m: – the number of the reaction
n: – the number of the gaseous component
M_i_: – molar mass of the i-th species, g mol^− 1^
r_ij_: – chemical production rate of the i-th species in j-th reaction, mol m^− 3^ s^− 1^
T_b_: – the temperature of the gas in bubbles, °C
T_bed_: – bed temperature, °C
T_CR1_: – the first critical temperature, °C
T_CR2_: – the second critical temperature, °C
T_e_: – the temperature of the emulsion phase, °C
u_br_: – rise bubble velocity, m s^− 1^
u_mf_: – superficial gas velocity at minimum fluidizing conditions, m s^− 1^
u_t_: – terminal velocity of a falling particle, m s^− 1^
V_air_: – air flow rate, L s^− 1^
V_LPG_: – fuel flow rate, L s^− 1^
x_i_: – mole fraction of the i-th species, -

## Greek symbols

${\upalpha }_{\mathrm {g}}^{\text {bc}}$: – heat transfer coefficient between the bubble phase and cloud-and-wake, W m^− 3^ deg^− 1^
${\upbeta }_{\mathrm {s}}^{\text {bc}}$: – mass transfer coefficient between the bubble phase and cloud, s^− 1^
ε_mf_: – void fraction in the emulsion phase at minimum fluidizing conditions, -
γ_b_: – volume fraction of solids inside a bubble
λ −: air excess coefficient, -
ρ_g_: – density of gas, kg m^− 3^
ρ_s_: – density of the solids, kg m^− 3^
τ: – time, s
